# Electrosprayed naringin-loaded microsphere/SAIB hybrid depots enhance bone formation in a mouse calvarial defect model

**DOI:** 10.1080/10717544.2019.1568620

**Published:** 2019-02-23

**Authors:** Xue Yang, Huthayfa N.S Almassri, Qiongyue Zhang, Yihui Ma, Dan Zhang, Mingsheng Chen, Xiaohong Wu

**Affiliations:** a Department of Prosthodontics, Stomatological Hospital of Chongqing Medical University, Chongqing, China;; b Chongqing Key Laboratory of Oral Diseases and Biomedical Sciences, Chongqing, China;; c Chongqing Municipal Key Laboratory of Oral Biomedical Engineering of Higher Education, Chongqing, China

**Keywords:** Burst release, osteogenesis, sucrose acetate isobutyrate, naringin, microsphere

## Abstract

The burst release of active osteogenic factors, which is not beneficial to osteogenesis, is commonly encountered in bone tissue engineering. The aims of this study were to prepare naringin-loaded microsphere/sucrose acetate isobutyrate (Ng-m-SAIB) hybrid depots, reduce the burst release of naringin (Ng), and improve osteogenesis. The morphology and size distributions of electrosprayed Ng-microspheres were characterized by scanning electron microscopy (SEM). The Ng-microspheres and Ng-m-SAIB depots were characterized by Fourier transform infrared spectroscopy (FTIR) and *in vitro* release studies. *In vitro* osteoblast-microsphere interactions and *in vivo* osteogenesis were assessed after implantation of Ng-m-SAIB depots. The addition of sucrose acetate isobutyrate (SAIB) to monodisperse Ng-microspheres did not cause a change in the chemical structure. The performances of the microspheres in osteoblast-microsphere interactions were better when the naringin content was 4% than when it was at 2% and 6%. On the first day following the loading of Ng-microspheres (2%, 4%, and 6%) into SAIB depots, the burst release was reduced dramatically from 70.9% to 6.3%, 73.1% to 7.2%, and 73.9% to 9.9%, respectively. In addition, after 8 weeks, the new bone formation rate in the calvarial defects of SD rats receiving Ng-m-SAIB was 53.1% compared to 21.2% for the control group and 16.1% for the microsphere-SAIB group. These results demonstrated that Ng-m-SAIB hybrid depots may have promise in bone regeneration applications.

## 1. Introduction

The criteria of ideal carriers for the sustained release of osteogenic drugs include biodegradability, biocompatibility, high drug loading, sustained release profile, and the ability to maintain the biological activity of osteogenic drugs (Zhao et al., 2015). From a pharmaceutical perspective, the sustained-release technology used in bone tissue engineering can play a significant role in promoting osteogenesis, prolonging the retention time of drugs, and minimizing the associated side effects caused by the burst release of high doses of these drugs (Liu et al., 2014).

Naringin (Ng), a significant ingredient in traditional Chinese medicine, has a regulatory effect on osteoblasts and osteoclasts (Chen et al., 2011; Ma et al., 2016; Xu et al., 2016). It is a hydrophilic drug, and its concentration can exert great influence on cell behavior, which means that a high concentration of Ng may be harmful to cells, whereas a low concentration may be ineffective (Tsui et al., 2008). Therefore, to facilitate sustained osseointegration, the prevention of initial burst release and facilitation of the controlled release of Ng warrant further study.

In previous studies on electrospun Ng-loaded polycaprolactone (PCL)/poly(ethylene glycol)-b-poly(ε-caprolactone) (PEG-b-PCL) formulations (Ji et al., 2014), PEG-b-PCL was applied to improve the encapsulation of Ng in order to reduce the burst release; however, the burst release remained at a level of 20% after 1 day. Similar results were obtained by other researchers, such as Singh et al. (2016), who demonstrated that scaffold-hydrogel hybrid constructs had over 30% burst release in the first day, and Yu et al. (2017), who reported that a collagen/metal-organic framework/naringin composite had a burst release reaching over 60% in the first day. The burst release of osteogenic drugs can be caused by many factors. One common factor is that most carriers are gelatin foam or collagen, which have properties such as significant porosity and a fast degradation rate (Hollinger et al., 1998; Browne et al., 2013; Ss et al., 2013; Cheng et al., 2016); another is that a majority of osteogenic drugs or proteins are hydrophilic and can easily diffuse into the surrounding aqueous medium, thereby causing the undesirable phenomenon of burst release (Ramazani et al., 2016).

The sucrose acetate isobutyrate (SAIB) depot is a promising injectable sustained-release system (Okumu et al., 2002) with biodegradability and biocompatibility (Reynolds & Chappel, 1998). Moreover, it is notable that unlike other types of injectable materials, such as gelatin or silk, the SAIB depot does not comprise animal-derived proteins, which may be associated with high related costs and disease (Cheng et al., 2016). Adding a minimal amount of solvent, such as ethanol, can significantly reduce the viscosity of SAIB. When the depot is delivered into the body, it is transformed from a fluid state into a semisolid as the solvent diffuses from the depot to the surrounding tissue, followed by slow drug release. However, an initial burst release may also occur when the concentration of the drug dissolved in the solvent is too high (Huang & Brazel, 2001). Lin et al. (2015) demonstrated that combining PLGA microspheres with SAIB depots could reduce the burst release of risperidone to 0.64% after 1 day.

Microspheres are one of the most commonly used drug delivery systems, and the most extensively used technique for the delivery of small hydrophilic drugs is the double emulsion method (Ramazani et al., 2016). However, this method is usually associated with problems such as broad particle size distribution and low entrapment efficiency.

Compared to microspheres prepared by other conventional techniques, electrosprayed microspheres have many advantages, such as improved uniformity or monodispersity, decreased agglomeration, high encapsulation efficiency and simplicity of preparation (Yao et al., 2016). The mode of drug release resulting from uniform electrosprayed microspheres may be more controlled and more stable than that of conventional drug administration routes (Valo et al., 2009; Bock et al., 2012). PCL, a biodegradable and FDA-approved polymer, is also commonly used in the preparation of electrosprayed microspheres with high monodispersity (Edlund & Albertsson, 2000). Moreover, the amphiphilic material PEG-b-PCL is used to improve the hydrophobicity of PCL and to better encapsulate Ng (Ji et al., 2014).

In this study, monodisperse electrosprayed Ng-loaded PCL/PEG-b-PCL microspheres were prepared, and their properties were characterized (morphology, size distribution, drug release and osteoblast-microsphere interactions). Moreover, these Ng-microspheres were incorporated into SAIB depots to achieve better controlled Ng release, and the bone regeneration potential of the fabricated Ng-m-SAIB hybrid depot was examined in SD rats with calvarial defects.

## 2. Methods and materials

### 2.1. Materials

Poly(ε-caprolactone) (PCL) (MW = 80,000), naringin (Ng), and sucrose acetate isobutyrate (SAIB) were obtained from Sigma-Aldrich (St. Louis, MO). Poly (ethylene glycol)-block-poly(ε-caprolactone) (PEG-b-PCL) (PEG MW = 2000; PCL MW = 2000) was from Jinan Daigang Biomaterial Co., Ltd. (Shandong, China). SD rats were purchased from the animal center of Chongqing Medical University. Alpha-modified Eagle’s medium (α-MEM, HyClone, USA), fetal bovine serum (FBS, Gibco, Australia), antibiotics (Sigma, USA) and trypsin-EDTA (Beyotime, China) were used in the cell culture of osteoblasts. Chloral hydrate (Sangon Biotech, China) was used for general anesthesia of SD rats. All chemicals used were analytically pure.

### 2.2. Preparation of naringin-loaded microspheres (Ng-microspheres)

PCL was dissolved in chloroform to obtain a 4% (w/v) solution. PCL and PEG-b-PCL were mixed at a weight ratio of 5:1 to obtain a PCL/PEG-b-PCL solution. Naringin was dissolved in acetone to prepare a homogenous solution. Then, a solution of Ng (0%, 2%, 4%, or 6% w/w relative to PCL) was added to the transparent PCL/PEG-b-PCL solution. The solutions were loaded in 5 ml syringes with 20 G needles. Next, microspheres were prepared using a single-nozzle electrospraying setup (Beijing Yongkang Leye Technology Development Co. Ltd., China). The electrospraying parameters were as follows: a constant flow rate (Q = 1 ml/h) and an applied voltage of 10 kV. The PCL/PEG-b-PCL microspheres were collected with aluminum foil at a distance of 20 cm. Microspheres collected on the aluminum foil were used for scanning electron microscopy (SEM), fourier transform infrared spectroscopy (FTIR), and drug release studies *in vitro*, in cell culture and *in vivo*. The electrosprayed microspheres were stored in desiccators for several days to remove the residual organic solvent.

### 2.3. Characterization of Ng-microspheres

The morphology of the Ng-microspheres was examined by SEM (S-3000N, HITACHI, Japan) at an accelerating voltage of 20 kV after gold coating. The average diameters of the electrosprayed microspheres were measured using ImageJ 2.0 (USA). The coefficient of variation (CV) was used to evaluate the monodispersity or uniformity of the Ng-microspheres and was calculated using the following formula:

$$\text{CV}\%=\frac{\text{Standard}\;\text{deviation}}{\text{Mean}\;\text{particle}\;\text{size}}\times 100\%$$

The determination of free Ng in the microspheres was performed by ultracentrifugation. Microspheres (10 mg, approximately 0.2 mg of Ng) were dissolved in pure water, mixed thoroughly for 15 s to form a sample solution, transferred to an ultracentrifuge tube, and then centrifuged at 13,000 rpm for 10 min. The supernatant was removed and measured at a wavelength of 283 nm via a UV-Vis spectrophotometer (ND-2000, Thermo Scientific, USA).

Drug encapsulation efficacy (EE) experiments were performed to determine the actual amount of Ng entrapped in the microspheres. The Ng-microspheres (10 mg) were added to 1 mL of absolute methanol, sonicated for 10 min, and then centrifuged at 15000 rpm for 20 min. The supernatant was analyzed as mentioned before. The EE of Ng was calculated using the following formula:

$$\text{Encapsulation}\;\text{efficacy}\;\left(\text{EE}\right)=\left(1-\frac{\text{free}\;\text{naringin}}{\text{amount}\;\text{of}\;\text{naringin}\;\text{in}\;\text{the}\;\text{spheres}}\right)\times 100\%$$

### 2.4. Preparation of naringin/SAIB (Ng-SAIB) and naringin-microsphere/SAIB (Ng-m-SAIB) hybrid depots

Prior to use, 0.2 mg of naringin was dissolved in an SAIB/ethanol (85/15, w/w) system to obtain Ng-SAIB depots. Similarly, approximately 10 mg of Ng-microspheres were dispersed into the SAIB solution by vortexing for 5 min to prepare Ng-m-SAIB depots. The prepared Ng-SAIB and Ng-m-SAIB depots had a final drug loading of 2 mg/g (M1-SAIB).

### 2.5. FTIR

FTIR (Thermo Scientific Nicolet iS5, USA) was used to qualitatively analyze the electrosprayed microspheres, Ng-m-SAIB hybrid depots and their compositions. The PCL samples were hot-pressed into membranes. The naringin, PEG-b-PCL and Ng-microsphere samples were observed by attenuated total reflection (ATR); and the SAIB depot, by the transmission method.

### 2.6. *In vitro* release

Approximately 100 mg of the Ng-m-SAIB depot and Ng-SAIB depot (equal to 0.2 mg naringin) were each injected into 1 ml of phosphate-buffered saline (PBS) solution (pH 7.4, 0.02% NaN_3_) in a 1.5 ml EP tube. Similarly, Ng-microspheres (equal to 0.2 mg naringin) were dispersed in a PBS solution. All the samples were incubated in a ZWY-110X30 reciprocal shaking water bath (Zhicheng Inc., China) at 37 °C. Samples were removed at each predetermined time point and replaced with 0.5 ml fresh medium (the microsphere suspensions were centrifuged at 13,000 rpm for 10 min before each time point). The samples were analyzed using a UV-Vis spectrophotometer. All experiments were carried out in triplicate.

### 2.7. Osteoblast-microsphere interactions

#### 2.7.1. Cell culture and CCK-8 assay

SD rats (2 ∼ 3 days old) were sacrificed by 75% alcohol, and their cranial bones were collected. The bones were cut into pieces and cultured for several days to obtain osteoblast cells. The osteoblast cells were cultured in α-MEM with 10% FBS and 100 U/ml antibiotics (penicillin-streptomycin-amphotericin) at 37 °C in 5% CO_2_ and replenished with microsphere suspensions every 2 days. The cytotoxicity of the Ng-microspheres (1 mg/mL) was estimated *in vitro* using a CCK-8 assay (Tongren, Japan). Osteoblast cells were seeded at a density of 2 × 10^4^ cells/well in 24-well plates and then incubated with microspheres at 37 °C after 24 h. After 1, 3, and 5 days of incubation, the cell culture medium was removed with an aspirator, and then 500 μL of fresh culture medium and 30 μL of CCK-8 were added to the 24 wells. After 3 h, aliquots of each sample were pipetted into a 96-well plate, and the absorbance was measured at 450 nm using an ELISA microplate reader (Thermo Scientific, USA).

#### 2.7.2. Cell differentiation by ALP

Osteoblast cells were seeded at a density of 2 × 10^4^ cells per well and then incubated with Ng-microspheres (1 mg/ml) at 37 °C. The expression level of alkaline phosphatase (ALP) in the cells was measured after 3, 7, and 10 days of incubation with a commercially available ALP colorimetric assay kit (Beyotime, Shanghai, China). The absorbance was measured at 405 nm using an ELISA microplate reader.

#### 2.7.3. Alizarin Red-S (ARS) staining

After 21 days of culture, cellular constructs were washed five times with PBS and fixed in 4% paraformaldehyde for 1 h. These constructs were then washed three times with distilled water and stained with 40 mM ARS (Solarbio, Beijing, China) for 30 min at room temperature. The staining of calcium nodules was observed by optical microscopy (Nikon, Japan). To assess the ARS staining, 10% cetylpyridinium chloride was added to rinse the dye as described previously (Liu et al., 2017).

### 2.8. Animal experiments

#### 2.8.1. Generation of a calvarial bone defect animal model

Adult male SD rats (13 ∼ 14 weeks old, *n* = 18) from the Institute of Experimental Animal Center of Chongqing Medical University were divided into the following three groups (*n* = 6): (1) control (empty defect), (2) blank microsphere-SAIB (m-SAIB) depot and (3) Ng-m-SAIB depot (M3-SAIB). All experimental procedures were approved by the Committee on the Ethics of Animal Experiments of Chongqing Medical University and were in compliance with the International Guiding Principles for Biomedical Research Involving Animals. All animals were anesthetized by intraperitoneal injection of 10% chloral hydrate (1 ml/250 g body weight). A 5 mm diameter full-thickness defect was created at both sides of the calvarium under low-speed drilling and normal saline irrigation. The defects were implanted with either the m-SAIB depot (about 0.1 g) or Ng-m-SAIB depot (about 0.1 g, 0.5 mg Ng) or were left unfilled as a control, and the incisions were closed with sutures. Penicillin was injected intraperitoneally (1 mg/kg) for 3 days following the operation.

#### 2.8.3. Microcomputed tomography (micro-CT)

At 2 and 8 weeks post-surgery, rats were sacrificed under overdose anesthesia. The harvested skulls were fixed in 10% neutral buffered formalin and analyzed by a micro-CT (Viva CT40, SCANCO Medical, Switzerland) at a resolution of 17.5 µm using an energy of 70 kV and 114 µA. The defect region was then identified by a cylindrical contour, and the new bone formation rate (BV/TV %) was calculated within this fixed volume of interest using SCANCO analysis software.

#### 2.8.4. Histologic and histomorphometric analyses

After micro-CT imaging, the entire skulls were decalcified with 10% ethylenediaminetetraacetic acid (EDTA) solution. Serial sections (5 µm thickness) were prepared along the coronal plane and stained with hematoxylin and eosin (HE) (Solabao, Beijing, China) and Masson trichrome (Bogu, Shanghai, China). Three central sections along the coronal plane were randomly selected for histomorphometric measurements.

#### 2.8.5. Immunohistochemistry (IHC)

The expression of the early osteogenic marker Runx-2 was evaluated at 2 weeks, while that of the late osteogenic marker osteocalcin (OCN) was detected at 8 weeks. Image analysis was performed by ImagePro Plus 6.0.

#### 2.8.6. Statistical analyses

The data were reported as the mean ± standard deviation (SD) for each experiment. SPSS 22.0 was used for data analysis. One-way analysis of variance (ANOVA) and the Student-Newman-Keuls (SNK-q) test were used for data analysis. For each test, *p* < .05 was considered statistically significant.

## 3. Results

### 3.1. Characterization of Ng-microspheres and Ng-m-SAIB depot

**Figure 1.**
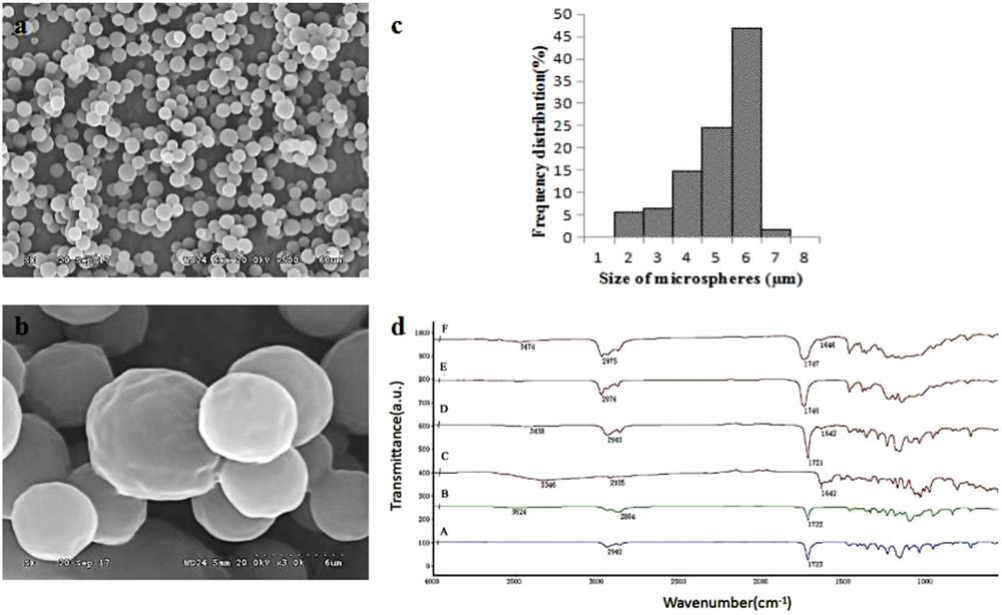
SEM image at 500 times (a) and 3000 times magnification (b), size distribution (c) of Ng-microspheres, and FTIR spectra (d) of PCL (A), PEG-b-PCL (B), naringin (C), electrosprayed PCL/PEG-b-PCL/naringin microspheres (Ng-microspheres) (D), SAIB (E), Ng-m-SAIB (F).

An SEM image of Ng-microspheres is shown in Figure 1. The microspheres were observed to have a slightly textured surface and to be almost spherical and monodispersed. The diameter of the microspheres was 5.52 ± 1.14 µm, and the polydispersity index was 21% (Figure 1(c)).

In this study, electrosprayed PCL/PEG-b-PCL microspheres were prepared with different amounts of Ng (0%, 2%, 4%, 6% w/w relative to PCL). For simplicity, the prepared Ng-microspheres are abbreviated as M0 (0%), M1 (2%), M2 (4%) and M3 (6%), respectively.

Following the loading of Ng into the microspheres, the amount of Ng in the microspheres increased as the Ng loading increased, and the amount of Ng (2%, 4%, 6%) in the microspheres was 87.9%, 90.3%, and 91.9%, respectively. However, the free Ng content increased with increasing Ng loading, and the encapsulation efficacy of the microspheres with different amounts of Ng (2%, 4%, 6%) was 63.5%, 64.3%, and 49.0%, respectively. These results demonstrated that when the amount of Ng increased to 6%, the amount of free Ng increased, and the encapsulation efficacy noticeably decreased.

Figure 1(d) shows the FTIR spectra of the Ng-microspheres, the Ng-m-SAIB depot and their composition. The PCL spectrum showed peaks of –CH2 stretching vibrations at 2942 cm^−1^ (2850–3000 cm^−1^) and –C=O stretching vibrations at 1723 cm^−1^ (1700–1800 cm^−1^) (Mu & Feng, 2002). PEG-b-PCL exhibited the characteristic peaks of PCL and had weak –OH stretching vibrations at 3524 cm^−1^, which agreed with previously published data (Hu et al., 2017). Ng showed the peaks of the C=O stretching vibrations at 1642 cm^−1^ (Feng et al., 2017) and –OH stretching vibrations at 3346 cm^−1^. SAIB showed the peaks of the –CH3 stretching vibrations at 2976 cm^−1^ and of the –C=O stretching vibrations at 1746 cm^−1^. These peaks were clearly observed in the spectra of both the Ng-microspheres and the Ng-m-SAIB depot without major shifting.

### 3.2. *In vitro* release of Ng-microspheres and Ng-m-SAIB depots

**Figure 2.**
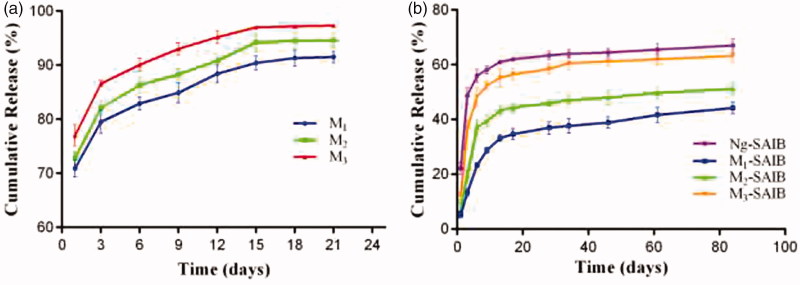
*In vitro* release from Ng-microspheres and SAIB-based depots. M1: naringin (2%)-loaded microspheres, M2: naringin (4%)-loaded microspheres and M3: naringin (6%)-loaded microspheres. Ng-SAIB: naringin (0.2 mg)-SAIB, M1-SAIB: naringin (2%)-loaded microspheres/SAIB, M2-SAIB: naringin (4%)-loaded microspheres/SAIB, M3-SAIB: naringin (6%)-loaded microspheres/SAIB. The results represent the average ± SD.

The *in vitro* release profiles of Ng from Ng-microspheres (M1, M2, M3) are shown in Figure 2(a). The release patterns of the Ng-microspheres were all characterized by an initial burst release (over 70%) on the first day. The burst release from M1 and M2 was near 80% over the 3 days, followed by a steady slow release stage until the 15th day, whereas the burst release from M3 was near 90% after 3 days.

The release patterns of the Ng-m-SAIB and Ng-SAIB depots are displayed in Figure 2(b). After Ng-microspheres (M1, M2, M3) were loaded into SAIB depots, the burst release on the first day was reduced dramatically from 70.9% to 6.3%, from 73.1% to 7.2%, and from 73.9% to 9.9%, respectively. By contrast, the Ng-SAIB depots still showed a clear initial burst release (up to 22.2%). After 6 days, the release from the Ng-m-depots (M1-SAIB, M2-SAIB, M3-SAIB) was 23.4%, 37.2%, and 48.3%, respectively, and that from the Ng-SAIB depot was 56%. Over time, the release from the SAIB-based depots was sustained and remained continuous until the 61st day. The linear fits of the naringin release curves from the SAIB depots demonstrated the presence of two release stages (Table 1). The slope represents the drug release rate, and the intercept in the first stage is related to the burst release according to the Higuchi equation (Yu et al., 2017).

**Table 1.** Evaluation of drug release kinetics of SAIB-based depot according to the Higuchi equation.

Depot	Ng-SAIB		M1-SAIB		M2-SAIB		M3-SAIB	
Stage (days)	1–3 day	6–61 day	1–6 day	9–61 day	1–6 day	9–61 day	1–6 day	9–61 day
Slope*	13.26	0.15	3.60	0.21	5.99	0.17	6.99	0.17
Intercept*	8.94	57.83	2.00	29.83	1.33	40.45	9.03	52.95
R	1.0000	0.7709	0.9977	0.8680	0.9999	0.8502	0.9008	0.8545

*The slope represented the drug release rate, the intercept in the first stage was related to the burst release according to the Higuchi equation.

### 3.3. Osteoblast-microsphere interactions

**Figure 3.**
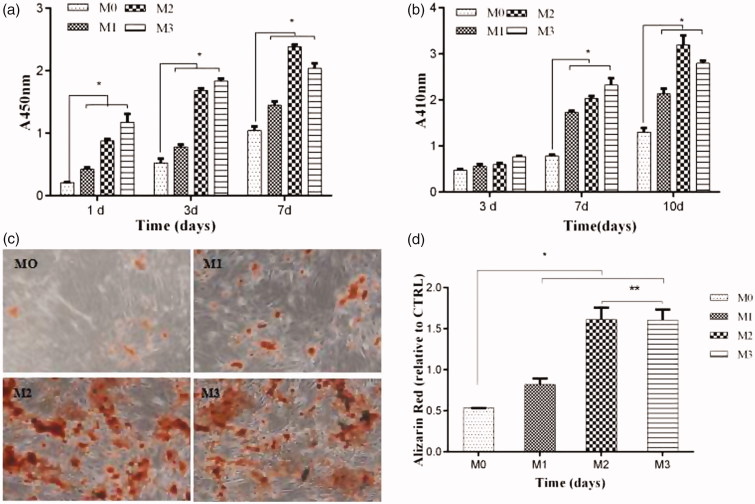
CCK-8 assay after 1, 3, and 7 days (a); ALP activity after 3, 7, and 10 days (b); ARS stains after 21 days (c); and the corresponding optical density of the dye solutions (d) of Ng-microspheres. M0: blank microspheres, M1: naringin (2%)-loaded microspheres, M2: naringin (4%)-loaded microspheres, and M3: naringin (6%)-loaded microspheres. The results represent the average ± SD, *p < .05, **p > .05; *n* = 3.

The proliferation of osteoblasts with Ng-microspheres (M0, M1, M2, M3) was evaluated by a CCK-8 assay after 1, 3, and 7 days (Figure 3a). The proliferation of osteoblasts for all the groups increased with time. The values of osteoblast proliferation for the M1, M2, and M3 groups were higher than those for the control group (M0) at all time-points (*p* < .05). Moreover, the values of osteoblast proliferation increased with increasing naringin concentration after 1 and 3 days; however, the values of osteoblast proliferation for M2 were higher than those for all other groups after 7 days.

The ALP activity of osteoblasts after 3, 7, and 10 days is shown in Figure 3(b). The ALP activity in the M1, M2, and M3 group was higher than that in the control group (M0) at all time-points (*p* < .05), except that no significant difference was found among the four groups after 3 days (*p* > .05). Moreover, the ALP activity increased with increasing naringin concentration after 3 and 7 days, whereas that in the M2 group was significantly higher than that in all other groups after 10 days.

Microscopy images of the stained microsphere-osteoblast hybrid after 21 days of incubation are shown in Figure 3(c). ARS nodules were observed in all groups, and the stains were more evident in the M2 and M3 groups than in the M1 and control groups. These phenomena were further confirmed by the results obtained using a dye solution and the corresponding optical density measurements at 485 nm (Figure 3(d)).

### 3.4. *In vivo* study

No suppuration or exposure of the material was observed in the defect sites during 2 and 8 weeks of healing.

#### 3.4.1. Micro-CT assessment of new bone formation

**Figure 4.**
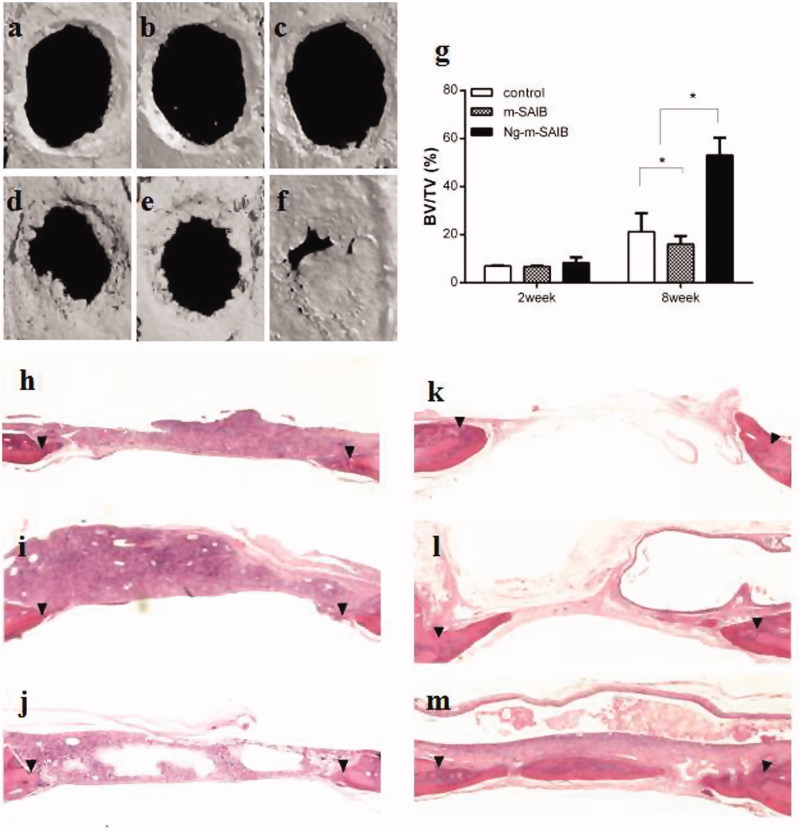
Reconstructed 3D images (a–f), statistical quantifications of the new bone formation rate (BV/TV %) (g), and representative HE images of different groups at 2 and 8 weeks postsurgery. a, d, h, k: control; b, e, i, l: m-SAIB; c, f, j, m: Ng-m-SAIB; a–c, h–j: after 2 weeks of surgery; d–f, k–m: after 8 weeks of surgery. The results represent the average ± SD, *p < .05. Black arrows represent the defect margin.

Representative 3D micro-CT images and the corresponding BV/TV data after 2 weeks and 8 weeks are shown in Figure 4. There was no significant difference observed in the 3 groups after 2 weeks (*p* > .05). After 8 weeks of bone defect healing, the Ng-m-SAIB group exhibited improved bone formation with BV/TV reaching 53.1%, while the BV/TV values of the control and m-SAIB groups were 21.2% and 16.1%, respectively.

#### 3.4.2. Histological assessment of new bone formation

After 2 weeks, limited or minimal new bone formation was observed along the margin of the defect in all the groups. The defect was occupied by connective tissue, in which a large number of inflammatory cells were observed, especially in the SAIB-based groups. After 8 weeks, the newly formed bone in the Ng-m-SAIB group was not only in the margin but also in the center of the defect, whereas defects from the control and m-SAIB groups were still mainly filled with connective tissue. In addition, no obvious inflammatory response was observed in the connective tissue. Bubble-like structures, comprising the unresorbed SAIB material, were observed in the SAIB-based groups (Figure 4).

**Figure 5.**
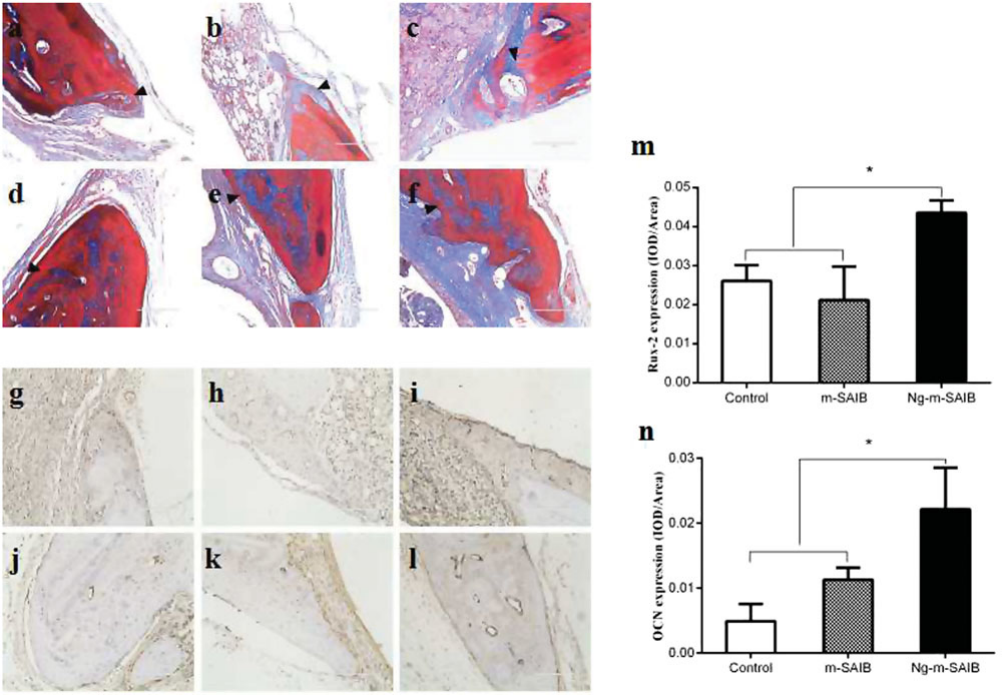
Results of Masson staining after 2 weeks (a–c) and 8 weeks (d–f) in each group, IHC staining for the osteogenic marker Runx-2 in each group after 2 weeks (g–i) and for the osteogenic marker OCN after 8 weeks (j–l) and the corresponding quantitative comparison of osteogenic expression of Runx-2 after 2 weeks (m) and OCN after 8 weeks (n). a, d, g, j: control; b, e, h, k: m-SAIB; c, f, i, l: Ng-m-SAIB. Scale bar, 100 µm; black arrows represent the defect margin, and the results represent the average ± SD, *p < .05, *n* = 3.

Masson staining of the defect margin in each group is shown in Figure 5. After 2 weeks, the new bone formed in the defects was rich in fiber and osteoblasts (Figure 5(a–c)). After 8 weeks, there were some osteoblasts on the edge of the new bone in the Ng-m-SAIB group (Figure 5(f)).

#### 3.4.3 Expression of runx-2 and OCN

The results of IHC staining for Runx-2 and OCN are shown in Figure 5. The expression levels of Runx-2 and OCN in the Ng-m-SAIB group were higher than those in the control and m-SAIB groups. This difference was also evident in the quantitative analysis of osteogenic expression (Figure 5(m,n)).

## 4. Discussion

This study focused on carriers with improved properties for the sustained release of Ng, which has shown good performance in stimulating bone formation (Chen et al., 2016). The morphology of the microspheres showed a slightly textured surface, which may be related to the fact that chloroform has a slightly lower boiling point of 61.2 °C (Bock et al., 2011). Previous *in vivo* studies showed that a textured surface of implant materials was preferred for cell adhesion (Place et al., 2009). From this point of view, the morphology of the electrosprayed microspheres was beneficial to the subsequent studies on osteogenesis. Moreover, the electrosprayed microspheres showed a narrow size distribution, consistent with previous studies (Yao et al., 2016).

The FTIR spectra of the drug-loaded microspheres and their combination with SAIB depots showed the mixed vibrational absorbance bands of their compositions with no major shifting. The FTIR results indicated that their compositions remained unchanged after applying a high electrical voltage and blending them with the SAIB depots.

Although the amount of Ng in the microspheres increased, the encapsulation efficiency decreased as the content of free Ng increased. When the Ng content increased to 6%, the encapsulation efficiency was reduced to 49.0%, which indicated that increasing the amount of Ng may lead to an increase in Ng distribution, primarily on the surface of microspheres, and a decrease in the content of Ng encapsulated in the microspheres. This finding could be explained by the fact that when the content of Ng increased, it was difficult for the dissolved Ng to diffuse toward the center of the electrosprayed droplets during solvent evaporation, and they were consequently deposited at the surface of the microspheres (Hong et al., 2008).

For instance, numerous studies have demonstrated a dose-dependent effect of Ng on increasing the proliferation and differentiation of UMR-106 cells (Wong & Rabie, 2006), MC3T3-E1 cells (Wu et al., 2008), bone mesenchymal stem cells (Peng-Zhang et al., 2009) and human amniotic fluid-derived stem cells (Liu et al., 2017). In this study, the results of the CCK-8 assay, ALP activity assay, and ARS stains were consistent, showing that when the content of Ng in the microspheres was 4%, the cell proliferation and osteogenic activity were better than those when the content was 2% and 6%. These results confirmed that Ng had a dose-dependent effect on improving the proliferation and osteogenic differentiation of osteoblasts. Therefore, it is particularly important to pursue carriers for the sustained release of naringin and sustained osteogenesis.

In the *in vitro* study, microspheres alone had a burst release close to 70% (Figure 2(a)) and had no improvement over analogous products, such as electrospun fibers, used in previous studies (Ji et al., 2014). The reason might be that the electrosprayed microspheres had a larger surface area and that the encapsulation of Ng by amphiphilic PEG-b-PCL was limited, which could also be illustrated by the finding that the encapsulation efficiency was not obviously increased by increasing the amount of Ng in this study. However, the burst release of naringin was effectively reduced when microspheres were loaded into the SAIB depot (Figure 2(b)). For the Ng-m-SAIB depots, the process of drug release involved double diffusion barriers: the drug was released from the microspheres to the SAIB depots and then released from SAIB depots to the medium (Reynolds & Chappel, 1998; Huang & Brazel, 2001; Okumu et al., 2002; Cheng et al., 2013; Lin et al., 2015). Compared to that of M1-SAIB and M2-SAIB, the burst release of M3-SAIB was slightly higher (approximately 12%), which was the result of the large amount of dissolved drug released from the surface of the M3 microspheres. Additionally, the release rate of M1-SAIB was slower than that of Ng-SAIB from day 1 to day 6 because the early release stage of Ng-m-SAIB was mainly dissolution-controlled (Lin et al., 2015). From 9 to 61 days, the M1-SAIB group continued to release slowly, while the release rate of Ng-SAIB was slightly slower (Table 1). This inconsistency was because SAIB is not degradable *in vitro*; thus, the release rate in the latter stage was mainly dependent on the residual drug in the depot (Lin et al., 2015).

The optimum concentration of naringin is different *in vivo* and *in vitro*. So the optimum concentration of naringin *in vitro* can only be used as a reference for experiments *in vivo*. *In vitro* release, the concentration of naringin in Ng-m-SAIB depot was lower than that of naringin in microspheres after SAIB depot was added. Therefore, although the cell proliferation and osteogenic activity of M2 were better than those of M1 and M3 *in vitro*, M3-SAIB was used *in vivo* experiments just as a preliminary experiment.

*In vivo* experiments showed no significant difference in the amount of bone formation among the 3 groups at 2 weeks post-surgery, which may be due to the prominent role of the early stage inflammatory reaction resulting from the operation, as well as the diffusion of solvents from the depot to the surrounding tissue. Previous studies have shown that SAIB is biocompatible and biodegradable, although inflammation may occur in the early stage of implantation (Lin et al., 2015). Eight weeks after surgery, the results demonstrated that the Ng-m-SAIB depot played an important role in promoting osteogenesis. After 8 weeks, the new bone formation in the Ng-m-SAIB group increased by 53.1%, while that in the control and m-SAIB groups showed a limited increase. In this study, the improved performance of the Ng-m-SAIB group may be attributed to the distinct osteogenic properties of Ng and the long-term sustained release behavior of Ng from the SAIB hybrid depot.

The osteogenic marker Runx-2 plays a crucial role in the early stage of osteoblast maturation (Komori et al., 1997), and the osteogenic marker OCN is usually expressed at the late stage of osteogenic differentiation (Miraoui et al., 2009). The two markers are closely related to ERK signaling, through which Ng enhances osteogenic differentiation (Wang et al., 2017). In this study, the expression levels of Runx-2 after 2 weeks and OCN after 8 weeks in the Ng-m-SAIB group were significantly higher than those in the control and m-SAIB groups, as shown in Figure 5. This result was consistent with a previous study (Yu et al., 2017), in which an Ng-loaded multifunctional collagen coating was shown to enhance osteogenic differentiation by promoting the expression of OCN and Runx-2.

## 5. Conclusion

Uniform Ng-loaded microspheres were successfully prepared, and the microspheres showed effective biocompatibility and osteogenic potential *in vitro*. Although burst release from the microspheres still existed, the Ng-m-SAIB depot showed improved controlled release performance *in vitro* and improved osteogenesis *in vivo* after 8 weeks. The above results indicate that the Ng-m-SAIB depot may be a promising sustained-release carrier for bone tissue engineering. Moreover, it should also be noted that the SAIB depot, as an injectable construct, had limited control over the shape of new bone formation (Cheng et al., 2016). There were other limitations to this study, such as the lack of a degradation rate or release profile *in vivo*. However, our research serves as a preliminary study of the potential for Ng-m-SAIB depots to enhance bone formation. The injection of SAIB depots could be used in more minimally invasive applications for the treatment of certain medical conditions, such as jawbone fractures and periapical lesions (Shamma et al., 2017), through injection into the defect point instead of open surgery.
